# Understanding primary care providers’ perceptions of cancer prevention and screening in a predominantly rural healthcare system in the upper Midwest

**DOI:** 10.1186/s12913-019-4872-9

**Published:** 2019-12-30

**Authors:** Daniel M. Saman, Kayla M. Walton, Melissa L. Harry, Stephen E. Asche, Anjali R. Truitt, Hillary A. Henzler-Buckingham, Clayton I. Allen, Heidi L. Ekstrom, Patrick J. O’Connor, JoAnn M. Sperl-Hillen, Jeanette Y. Ziegenfuss, Joseph A. Bianco, Thomas E. Elliott

**Affiliations:** 1 Essentia Institute of Rural Health, 502 E. Second Street, Duluth, MN 55805 USA; 2 HealthPartners Institute, 3311 E. Old Shakopee Road, Bloomington, MN 55425 USA; 3 Essentia Health – Ely Clinic, 300 W. Conan Street, Ely, MN 55731 USA

**Keywords:** Cancer prevention, Cancer screening, Clinical decision support, Electronic medical record, Primary care provider

## Abstract

**Background:**

Cancer is the leading cause of death in the United States, with the burden expected to rise in the coming decades, increasing the need for effective cancer prevention and screening options. The United States Preventive Services Task Force has suggested that a shared decision-making process be used when clinicians and patients discuss cancer screening. The electronic medical record (EMR) often provides only reminders or alerts to primary care providers (PCPs) when screenings are due, a strategy with limited efficacy.

**Methods:**

We administered a cross-sectional electronic survey to PCPs (*n* = 165, 53% response rate) at 36 Essentia Health primary care clinics participating in a large, National Cancer Institute-funded study on a cancer prevention clinical decision support (CDS) tool. The survey assessed PCP demographics, perceptions of the EMR’s ability to help assess and manage patients’ cancer risk, and experience and comfort level discussing cancer screening and prevention with patients.

**Results:**

In these predominantly rural clinics, only 49% of PCPs thought the EMR was well integrated to help assess and manage cancer risk. Both advanced care practitioners and physicians agreed that cancer screening and informed discussion of cancer risks are important; however, only 53% reported their patients gave cancer screening a high priority relative to other health issues.

**Conclusions:**

The impact of EMR-linked CDS delivered to both patients and PCPs may improve cancer screening, but only if it is easy to use and saves PCPs time.

## Introduction

Cancer is emerging as the leading cause of death in developed countries [1]. In 2012, one in four deaths was due to cancer in the United States [2]. Risk of cancer increases with age, and due to the aging population, the burden of cancer is expected to increase worldwide in the next few decades [1, 2]. Thus, many experts, care delivery systems, and the International Agency for Research on Cancer are pushing for more active cancer prevention and screening options to reduce the growing burden of cancer.

Existing preventive methods, when used, are effective in reducing cancer risk. Tobacco use cessation, a healthy lifestyle, and healthy eating have all been shown to effectively reduce the risk of cancer [1]. For several common cancers, screening does lead to higher survival rates and less overall cancer burden [3]. For example, the updated United States Preventive Services Task Force (USPSTF) recommendations for colon cancer screening have significantly improved rates of screening and decreased the incidence of colorectal cancer in different populations [4].

However, cancer screening rates are far from optimal, and a simple recommendation for screening may not be enough to motivate patients to get screened, or patients may not be following through with testing [5]. Preliminary data from 2012 to 2014 among eligible Essentia Health patients aged 11–80 with two or more primary care visits within 36 months showed about two-thirds are up to date on colorectal cancer screening, two-thirds up to date on breast cancer screening, 54% up to date on cervical cancer screening, and 5% of males aged 11–26 and 20% of females are up to date on HPV vaccination. The USPSTF has suggested that a shared decision-making (SDM) process be used when discussing screening options [6]. SDM engages the patient as an informed partner to ensure that decisions reflect unique health needs and preferences. Provider-patient interaction plays a critical role in increasing screening adherence [3]. When patients are engaged in the decision-making process, they are more likely to take ownership over the course of action [7]. In addition, SDM has been shown to improve overall patient health and reduce health care costs [8].

One method for improving the quality of recommendations for cancer screening is use of the electronic medical record (EMR) to offer clinical decision support (CDS) tools. CDS tools are electronic systems that attach reminders and alerts to patients’ charts when they need certain types of care [9]. They are designed to both help improve direct clinical decision making and address gaps in health care [9–11]. While great emphasis has been placed on CDS tools, limited evidence supports their efficacy. In a 2012 meta-analysis by Bright et al., CDS tools were effective in increasing orders for preventive care services. However, many of these interventions used tools developed for only a few clinics, were not implemented systemwide, or were tied directly to the EMR. Indeed, benefits of CDS are limited, largely due to how it is implemented [11–13]. In a recent review by Rashanov et al. [13], the authors analyzed 162 randomized trials of CDS tools. Of these trials, only 58% successfully improved patient outcomes or provider care. CDS tools were significantly more likely to succeed if they gave advice to both providers and patients, required providers to explain why they overrode an alert, were developed by the study authors rather than a third party, and involved both the provider and the patient.

Despite existing CDS tools and the noted benefits of SDM, primary care providers (PCPs) often fail to include patients in the decision-making process for cancer screening [14–17]. There are often multiple or even conflicting recommendations for optimal care, and many patients are not well informed when making cancer screening decisions [18]. When discussion of cancer screening does occur, patients are often informed of the benefits—but not the risks. In their survey, Hoffman et al. [18] found that less than half of patients who had recently undergone cancer screening were able to correctly answer even one question about it. This suggests a serious gap in care delivery that could compromise a patient’s involvement in SDM. However, a study by Bryan et al. [19] suggested that educating PCPs about updated breast cancer screening recommendations and options increased their knowledge and changed their recommendations. The authors also noted that PCPs’ attitudes toward SDM and their comfort with discussing breast cancer screening significantly increased post-intervention. It appears that PCPs’ lack of SDM may be due in part to lack of education and comfort, but few studies have addressed this gap.

This survey was conducted as part of our larger National Cancer Institute-funded cancer CDS study to understand providers’ perceptions regarding cancer prevention prior to the implementation of our EMR-based CDS. The aims of this exploratory survey were threefold: 1) assess PCPs’ opinions about current EMR and cancer prevention CDS tools; 2) assess PCPs’ knowledge of current cancer screening and prevention recommendations; and 3) identify strategies that could narrow observed gaps in cancer prevention and screening by physicians and advanced care practitioners.

## Methods

### Study participants

Study participants included 335 PCPs practicing in at least one of 36 Essentia Health primary care clinics participating in a cancer prevention and screening CDS randomized controlled trial that included 24 intervention clinics and 12 control clinics. Essentia Health is a predominantly rural, upper Midwestern, integrated health care system. The survey was administered before implementation of a cancer prevention and screening CDS in the 24 intervention clinics. PCPs included physicians (family practice or internal medicine), advanced care practitioners (adult, pediatric, family, or geriatric), and physician assistants who provide ongoing care for 25 or more patients who met study eligibility criteria or worked 50% or more effort as a PCP. A follow-up survey focusing on CDS usage and SDM is currently being planned to determine whether perceptions on cancer prevention have changed following usage of the CDS.

### Survey instrument

The survey instrument queried PCPs in areas such as demographics, views on EMR-based CDS, tobacco cessation, weight management, risk calculators, SDM between patients and PCPs, SDM tools for cancer prevention and screening, and medical group commitment and preparedness to maximize cancer prevention interventions. Survey questions were developed by the study team (Additional file 1) or adapted from validated instruments used in the National Survey of Primary Care Physicians’ Recommendations & Practice for Breast, Cervical, Colorectal, & Lung Cancer Screening [20] and the System Usability Scale (SUS) [21].

### Data collection

The survey was administered electronically from November 2, 2017 to January 24, 2018. PCPs in the 36 study clinics were initially emailed an invitation to take part in the survey signed by the site principal investigator. PCPs were then emailed as many as seven reminders if they did not complete the survey. Survey completion implied PCP consent. This study was reviewed and approved by the Essentia Health Institutional Review Board.

### Data analysis

Descriptive and bivariate data analyses were conducted in SAS v. 9.4 [22] by SA. Bivariate tests of association compared responses from physicians and advanced care practitioners (physician assistants and advanced care practitioners). These included cross-tabulations reporting chi-square (*χ*^*2*^), Fisher’s exact test when cell counts were < 5, and Cramer’s *V* (*φ*_*c*_) and Phi (*φ*) for assessing the strength of associations with nominal data. Categorical responses with more than two options were recoded into binary variables for analysis (e.g., “Sometimes/Never” and “Always/Usually”; “Very effective” and “Somewhat/Not effective/Don’t know”; “Very/Somewhat uncomfortable” and “Somewhat/Very comfortable”; “Somewhat/Not helpful” and “Very helpful”; “Strongly/Somewhat agree” and “Neither agree nor disagree/Strongly/Somewhat disagree”; “Medium/Low priority” and “High priority”; “Somewhat/Not prepared” and “Very prepared”). Tests were two-tailed, with an alpha of .05.

## Results

### Descriptive data

Of the 335 potential study participants, 312 had functioning email addresses signifying they were still employed by Essentia Health. Of those 312 PCPs, 165 (53%) returned either a fully or partially completed survey. As shown in Tables 1, 62% of respondents were women, advanced care practitioners were more likely to be women than were physicians, *χ*^*2*^ (1, *n* = 164) = 42.24, *p* < .001, *φ* = 0.51, and physicians were significantly more likely to be older than advanced care practitioners, *χ*^*2*^ (3, *n* = 164) = 19.88, *p* < .001, *φ*_*c*_ = 0.35. Physicians were also significantly more likely to have been in practice longer than advanced care practitioners, with 68% of physicians in practice 11 years or more, compared with 32% of advanced care practitioners, *χ*^*2*^ (2, *n* = 165) = 27.58, *p* < .001, *φ*_*c*_ = 0.41.

**Table 1:** Respondent Demographics

	Provider Type		
	*n*	AP/PA *n* (%)	Physician *n* (%)
Age range (years)^***^	164		
≤ 34		24 (35)	10 (10)
35–44		18 (26)	20 (21)
45–54		12 (18)	23 (24)
≥ 55–64		14 (20)	43 (45)
Years in practice^***^	165		
≤ 5		37 (54)	16 (16)
6–10		9 (13)	15 (15)
≥ 11		22 (32)	66 (68)
Days/week seeing patients	165		
0–2		7 (10)	11 (11)
3		6 (9)	20 (21)
4		30 (44)	39 (40)
5		25 (37)	27 (28)
Sex^***^	165		
Male		6 (9)	57 (59)
Female		62 (91)	40 (41)

*AP/PA* Advanced care practitioner/physician assistant

^***^P < .001.

### Cancer screening and prevention

When asked about health priorities, 53% of PCPs reported their patients give cancer screening a high priority in relation to other health services, but physicians (63%) reported their patients were significantly more likely to place high priority on cancer screening than those of advanced care practitioners (40%) (*χ*^*2*^ (1, *n* = 165) = 8.63, *p* = .003, *φ* = 0.23). PCPs were also asked about having adequate time to discuss different cancer screening options (Table 2), including cancer prevention using the human papillomavirus (HPV) vaccination (Table 3). Most PCPs reported that they do not always have enough time to discuss screening or HPV vaccination with patients (Tables 2 and 3). Seventy-seven percent of PCPs said that patients sometimes do not know about the HPV vaccine, and most (93%) noted that patients usually or sometimes do not perceive HPV as a serious health threat (Table 3). However, no significant differences were seen between physicians and advanced care practitioners.

**Table 2:** Provider Perspectives on Time to Discuss Cancer Screening

How often do you have enough time to discuss the following with your patients?:	Response Options				
	*n*	Never *n* (%)	Sometimes *n* (%)	Usually *n* (%)	Always *n* (%)
Breast Cancer Screening	153	22 (15)	87 (57)	36 (23)	8 (5)
Colon Cancer Screening	153	23 (15)	104 (68)	22 (14)	4 (3)
Lung Cancer Screening	145	7 (5)	96 (66)	35 (24)	7 (5)
Cervical Cancer Screening	148	38 (26)	93 (63)	13 (9)	4 (3)

**Table 3:** Provider Perspectives on Human Papillomavirus (HPV) Vaccination

When you talk to patients about HPV vaccination, how often do you encounter the following?	Response Options				
	*n*	Always *n* (%)	Usually *n* (%)	Sometimes *n* (%)	Never *n* (%)
Not having enough time to discuss HPV vaccination with my patients	146	5 (3)	19 (13)	92 (63)	30 (21)
Patients who do not want to discuss HPV vaccination	146	1 (1)	34 (23)	100 (68)	11 (8)
Patients who are unaware of HPV vaccination	145	1 (1)	19 (13)	111 (77)	14 (10)
Patients who do not perceive HPV as a serious health threat	143	4 (3)	63 (44)	70 (49)	6 (4)

When asked about preparedness, 63% of PCPs reported feeling very prepared to prioritize cancer risk factors and screening and discuss with their patients, compared to 37% feeling somewhat or not prepared. Overall, 93% of PCPs said they were very or somewhat comfortable discussing increased cancer risk with overweight or obese patients. However, advanced care practitioners (13%, *n* = 9) were significantly more likely to be very or somewhat uncomfortable discussing increased risk of cancer with overweight or obese patients than physicians (3%, *n* = 3; *p* = .03, *φ* = 0.19). There were no significant differences between groups regarding comfort level discussing increased cancer risk with patients who smoke. Overall, 98% of PCPs felt very or somewhat comfortable discussing this with patients who smoke (not reported in tables).

### Electronic medical record

As shown in Table 4, about 57% of PCPs somewhat or strongly agreed the EMR was easy to use for helping assess and manage cancer risk. About half (49%) somewhat or strongly agreed the EMR was well integrated to help assess and manage cancer risk, and 66% (*n* = 107) either disagreed or were neutral (somewhat disagree, strongly disagree, neither agree nor disagree) with the notion the EMR was easy to learn to use to help manage cancer risk. No significant differences were observed between physicians and advanced care practitioners.

**Table 4:** Provider Perspectives on Electronic Medical Record (EMR) Decision Support Effectiveness

Survey Questions^a^	Response Options					
	*n*	Strongly disagree *n* (%)	Somewhat disagree *n* (%)	Neither agree nor disagree *n* (%)	Somewhat agree *n* (%)	Strongly agree *n* (%)
Our EMR decision support is easy to use for helping me assess and manage a patient’s cancer risk.	163	7 (4)	43 (26)	21 (13)	78 (48)	14 (9)
The various functions in our EMR decision support are well integrated for helping to assess and manage a patient’s cancer risk.	163	14 (9)	37 (23)	31 (19)	69 (42)	12 (7)
Most providers easily learn how to use the EMR decision support to help them manage a patient’s cancer risk.	161	11 (7)	54 (34)	42 (26)	44 (27)	10 (6)
Our EMR decision support is awkward/cumbersome to use for helping assess and manage a patient’s cancer risk.	163	9 (6)	29 (18)	50 (31)	56 (34)	19 (12)

^a^Questions adapted from the SUS (Brooke, 1996)

When asked about different functions of the EMR, most PCPs (68%) noted that the EMR did not alert them when cervical cancer screening was due (Table 5). Thirty-seven percent also said they were not alerted when lung cancer screening was due, and 26% said they were not alerted when an HPV vaccination was due. Table 5 also indicates that most PCPs did not think the EMR supported printouts of useful information to help patients identify a preferred screening method or could classify patients based on individual cancer risk scores.

**Table 5:** Provider Perspectives on the Electronic Medical Record (EMR) for Cancer Prevention and Screening

Survey Questions	Response Options	
	Yes *n* (%)	No *n* (%)
The EMR alerts me that this action is due.		
Breast Cancer Screening	148 (95)	8 (5)
Cervical Cancer Screening	50 (32)	105 (68)
Colorectal Cancer Screening	139 (91)	14 (9)
Lung Cancer Screening	97 (63)	56 (37)
HPV Vaccination	113 (74)	40 (26)
The EMR makes it easy for me to order the needed service.		
Breast Cancer Screening	130 (84)	24 (16)
Cervical Cancer Screening	92 (60)	61 (40)
Colorectal Cancer Screening	128 (84)	25 (16)
Lung Cancer Screening	98 (66)	51 (34)
HPV Vaccination	113 (76)	35 (24)
The EMR enables me to print out materials that help patients identify their preferred screening method.		
Breast Cancer Screening	49 (33)	102 (67)
Cervical Cancer Screening	42 (28)	107 (72)
Colorectal Cancer Screening	55 (37)	93 (63)
Lung Cancer Screening	47 (32)	101 (68)
HPV Vaccination	51 (35)	94 (65)
The EMR makes it easy to calculate cancer risks for individual patients.		
Breast Cancer Screening	12 (8)	140 (92)
Cervical Cancer Screening	11 (7)	141 (93)
Colorectal Cancer Screening	16 (11)	137 (89)
Lung Cancer Screening	29 (19)	122 (81)
HPV Vaccination	19 (12)	133 (87)

*HPV* Human papillomavirus

## Discussion

Few studies have assessed PCPs’ attitudes about cancer screening and prevention in predominately rural areas. As noted elsewhere, engaging patients through cancer SDM with PCPs is challenging, particularly given current time constraints in primary care [23, 24]. Although 37% of PCPs were not very prepared to discuss cancer risk factors and screening with patients, less than half (49%) said that the EMR was well integrated to help assess and manage cancer risk. Moreover, only 57% of PCPs said that the EMR was easy to use for helping assess and manage cancer risk**.** While providers generally reported the EMR does well around ordering screenings, the EMR does not calculate individual cancer risk well, and does not allow for printing materials to assist patients in making decisions. These data are somewhat mixed, yet point to the need for a more useful and practical EMR that better helps PCPs make decisions and assess cancer risk for patients.

Only 53% of respondents said that their patients give cancer screening a high priority in relation to other health services, and this percentage was even lower in advanced care practitioners. This finding could be due to several issues we identified in key informant interviews [24] and through continued engagement with our intervention clinics: lack of time for patients and/or providers to discuss cancer prevention; and patients visiting for acute reasons. Most PCPs also reported that they often do not have enough time to discuss screening or HPV vaccination with patients. This points to the challenges associated with improving rates of cancer prevention and screening in a high-pressure, time-constrained primary care environment [25, 26] and supports the need to optimize the EMR to more easily address cancer prevention and screening needs.

We observed few statistically significant differences between physicians and advanced care practitioners in this study. Of note, compared with advanced care practitioners, physicians reported their patients were significantly more likely to place high priority on cancer screening, and physicians were more comfortable discussing increased risk of cancer with overweight or obese patients. The relative similarities in perceptions between PCPs and advance care practitioners in other areas may be due to statewide initiatives such as Minnesota Community Measurement [27], quality standards for health care systems such as measures of breast and colorectal cancer screening, and Essentia Health’s common approach to quality improvement and endorsement of statewide and national quality measures.

Factors that limit the interpretation of these survey data include nonresponse bias, social desirability responses, and missing data from survey noncompletion. Although we adapted the SUS by reducing the number of questions, the SUS-based questions asked in this study provide useful information on PCP perceptions of attitudes towards cancer prevention and screening and satisfaction with current EMR-linked cancer CDS. Results of the survey might differ in other care delivery systems and would be expected to vary over time. The survey results are also limited in generalizability, as we were only interested in understanding perceptions of PCPs within Essentia Health.

Strengths of the study included administering a confidential electronic survey, achieving a high survey response rate, and inviting all PCPs who provide ongoing primary care for 25 or more patients to take the survey. A post-intervention implementation PCP survey is planned for future study years to allow assessment of changes in PCP opinions over time following the use of a developed CDS targeted to improve cancer screening and prevention known as the Cancer Prevention Wizard study [24, 28].

## Conclusion

One of a health care organization’s many responsibilities is to improve patients’ lives through the prevention and early detection of cancer. This burden and opportunity have largely been placed on the shoulders of PCPs, who must routinely prioritize and juggle competing patient and clinic demands. As cancer screening and prevention technology improve, risk prediction based on biomarkers will ultimately lead to personalized cancer prevention and detection strategies. As science moves in this direction, the importance of EMR-linked CDS and the need for patients to be well-informed about clinical options will increase dramatically. Although most PCPs said that cancer prevention and screening is important, it is clear that algorithmic personalization of clinical options will become an essential feature of adequate care. The data presented here underscore that the current state of affairs is untenable and suggest that development of more effective and efficient models for cancer prevention CDS and SDM is urgently needed.

## Supplementary information

**Additional file 1:.** Cancer prevention provider survey. Provider survey questions used in this study.

## Data Availability

The data analyzed during the current study are available from the corresponding author on reasonable request.

## Abbreviations

CDS: Clinical Decision Support
EMR: Electronic Medical Record
PCP: Primary Care Provider
SDM: Shared Decision-Making
SUS: System Usability Scale
USPSTF: United States Preventive Services Task Force
