# Supplementary material for: Understanding primary care providers’ perceptions of cancer prevention and screening in a predominantly rural healthcare system in the upper Midwest
Source: BMC Health Serv Res. 2019 Dec 30;19:1019. doi: 10.1186/s12913-019-4872-9 (PMC6937782; doi:10.1186/s12913-019-4872-9)
Supplement: Supplementary file 1 — Additional file 1:. Cancer prevention provider survey. Provider survey questions used in this study. [file 12913_2019_4872_MOESM1_ESM.pdf]

Essentia Health Cancer Prevention Survey

Thank you for taking the time to complete this important survey. As you know, many of our patients at Essentia are at high risk for developing cancer. To better understand and guide future care practices, we would like to know your experience in providing care to these patients.

1. Please tell us your current role within your 'home' primary care clinic? (Please check only one)

◯ Nurse Practitioner
◯ Physician Assistant
◯ Family practice physician
◯ Internal medicine physician
◯ Registered Nurse
◯ Other

Please specify: (If endorsed "Other" above)

____

2. How long have you been in practice? (do not include training and residency)

◯ less than 1 year
◯ 1-5 years
◯ 6-10 years
◯ 11 or more years

3. On average, how many days a week do you see patients in the clinic?

◯ 0
◯ 1
◯ 2
◯ 3
◯ 4
◯ 5
◯ 6
◯ 7

4. What is your age?

◯ 34 years old or younger
◯ 35-44 years old
◯ 45-54 years old
◯ 55-64 years old
◯ 65 years old or older

5. Are you male or female?

◯ Male
◯ Female

6. How comfortable are you discussing the increased risk of cancer for patients...

	Very comfortable	Somewhat comfortable	Somewhat uncomfortable	Very uncomfortable
Who smoke?	◯	◯	◯	◯
Who are overweight or obese?	◯	◯	◯	◯

7. How well prepared do you feel to prioritize cancer risk factors and screening and discuss them with your patients?

◯ Very prepared
◯ Somewhat prepared
◯ Not prepared

8. In relation to other health issues, what level of priority do your patients give cancer screening?

◯ High priority
◯ Medium Priority
◯ Low priority

9. For each of the following statements, select the response that best describes your reactions to your EMR's ability to help assess and manage patient cancer risk. By "EMR decision support" we mean any prompts, reminders, or other clinical decision support that is directed to cancer-related risk and prevention.

	Strongly disagree	Somewhat disagree	Neither agree nor disagree	Somewhat agree	Strongly agree
a. Our EMR decision support is easy to use for helping me assess and manage a patient's cancer risk.	○	○	○	○	○
b. The various functions in our EMR decision support are well integrated for helping to assess and manage a patient's cancer risk.	○	○	○	○	○
c. Most providers easily learn how to use the EMR decision support to help them manage a patient's cancer risk.	○	○	○	○	○
d. Our EMR decision support is awkward/ cumbersome to use for helping assess and manage a patient's cancer risk.	○	○	○	○	○

10. Has your clinic used the following strategies to improve cancer prevention in your population?

a. Provided information and skills-training to staff related to improved cancer prevention.

◯ Yes
◯ No

How helpful was the information and training that was provided? (If endorsed "Yes" above)

◯ Very helpful
◯ Somewhat helpful
◯ Not helpful

b. Provided me with measurement reports on provider or clinic performance for cancer prevention.

◯ Yes
◯ No

How helpful were the reports? (If endorsed "Yes" above)

◯ Very helpful
◯ Somewhat helpful
◯ Not helpful

c. Cancer prevention strategies have been implemented to improve clinician efficiency to assess and manage cancer risk.

◯ Yes
◯ No

How helpful was this strategy? (If endorsed "Yes" above)

◯ Very helpful
◯ Somewhat helpful
◯ Not helpful

11. Considering all the priorities your clinic has over the next year (e.g., EMR, financial goals, quality improvement of various conditions, MD recruitment), what is the priority that your clinic has placed on improving cancer prevention?

◯ 1 (Not a priority)
◯ 2
◯ 3
◯ 4
◯ 5 (Medium priority)
◯ 6
◯ 7
◯ 8
◯ 9
◯ 10 (Highest priority of all)

12. Select "yes" or "no" for the following questions on the EMR your clinic uses for screening of breast, cervical, colorectal, or lung cancers or HPV vaccination.

a. The EMR alerts me that this action is due.

	Yes	No
Breast Cancer Screening	◯	◯
Cervical Cancer Screening	◯	◯
Colorectal Cancer Screening	◯	◯
Lung Screening	◯	◯
HPV Vaccination	◯	◯

b. The EMR makes it easy for me to order the needed service.

	Yes	No
Breast Cancer Screening	◯	◯
Cervical Cancer Screening	◯	◯
Colorectal Cancer Screening	◯	◯
Lung Screening	◯	◯
HPV Vaccination	◯	◯

c. The EMR enables me to print out materials that help patients identify their preferred screening method.

	Yes	No
Breast Cancer Screening	◯	◯
Cervical Cancer Screening	◯	◯
Colorectal Cancer Screening	◯	◯
Lung Screening	◯	◯
HPV Vaccination	◯	◯

d. The EMR makes it easy to calculate cancer risks for individual patients.

	Yes	No
Breast Cancer Screening	◯	◯
Cervical Cancer Screening	◯	◯
Colorectal Cancer Screening	◯	◯
Lung Screening	◯	◯
HPV Vaccination	◯	◯

13. At preventive care visits, how often do you use a cancer risk calculation for any of the following?

	Always	Usually	Sometimes	Never
Breast cancer (e.g., BCRAT)	○	○	○	○
Colorectal cancer (e.g., CRCAT)	○	○	○	○
Lung cancer (e.g., CDC/AHRQ)	○	○	○	○

Breast Cancer Screening

14. When you talk to patients about breast cancer screening, how often do you encounter the following?

	Always	Usually	Sometimes	Never
a. Not having enough time to discuss screening with my patients	◯	◯	◯	◯
b. Patients who do not want to discuss breast cancer screening	◯	◯	◯	◯
c. Patients who have difficulty understanding the information I present about breast cancer screening	◯	◯	◯	◯
d. Patients who are unaware of breast cancer screening	◯	◯	◯	◯
e. Patients who do not perceive breast cancer as a serious health threat	◯	◯	◯	◯
f. Patients who cannot afford or lack adequate insurance coverage for breast cancer screening	◯	◯	◯	◯
g. Other patient barriers to breast cancer screening I see	◯	◯	◯	◯

Please specify: (If endorsed "Always", "Usually", or "Sometimes" for g. Other above)

15. How comfortable are you advising your patients on breast cancer screening options?

◯ Very comfortable
◯ Somewhat comfortable
◯ Somewhat uncomfortable
◯ Very uncomfortable

Colorectal Cancer Screening

16. Consider the last patient you saw regarding colorectal cancer screening. For each of the following six statements related to the decision-making in this consultation, please select the response that best describes your actions with this patient.

	Strongly disagree	Somewhat disagree	Neither agree nor disagree	Somewhat agree	Strongly agree
a. I told my patient that there are different screening options (e.g. FIT, Colonoscopy).	◯	◯	◯	◯	◯
b. I explained the advantages and disadvantages of the screening options to my patient.	◯	◯	◯	◯	◯
c. I helped my patient understand all the information.	◯	◯	◯	◯	◯
d. I asked my patient which screening option he/she prefers.	◯	◯	◯	◯	◯
e. My patient and I selected a screening option together.	◯	◯	◯	◯	◯
f. My patient and I reached an agreement on how to proceed.	◯	◯	◯	◯	◯

17. How effective do you believe the following screening procedures are in reducing colorectal cancer mortality in average-risk patients aged 50 years and older?

How effective is...

	Very effective	Somewhat effective	Not effective	Don't know
a. Fecal immunochemical tests (FIT) (Polymedco OC-Light® S FIT or Polymedco OC-Auto® FIT)	◯	◯	◯	◯
b. Colonoscopy	◯	◯	◯	◯
c. Fecal DNA testing (Cologuard®)	◯	◯	◯	◯

18. How often did you recommend colorectal cancer screening tests other than colonoscopy to your asymptomatic, average-risk patients during the past 12 months?

◯ Always
◯ Usually
◯ Sometimes
◯ Never

19. When you talk to patients about colorectal cancer screening, how often do you encounter the following?

	Always	Usually	Sometimes	Never
a. Not having enough time to discuss screening with my patients	◯	◯	◯	◯
b. Patients who do not want to discuss colorectal cancer screening	◯	◯	◯	◯
c. Patients who have difficulty understanding the information I present about colorectal cancer screening	◯	◯	◯	◯
d. Patients who are unaware of colorectal cancer screening	◯	◯	◯	◯
e. Patients who do not perceive colorectal cancer as a serious health threat	◯	◯	◯	◯
f. Patients who cannot afford or lack adequate insurance coverage for colorectal cancer screening	◯	◯	◯	◯
g. Other	◯	◯	◯	◯

Please specify: (If endorsed "Always", "Usually", or "Sometimes" for g. Other above)

20. How comfortable are you advising patients about selecting a particular method of colorectal cancer screening?

◯ Very comfortable
◯ Somewhat comfortable
◯ Somewhat uncomfortable
◯ Very uncomfortable

Lung Cancer Screening

21. Consider the last patient you saw who was a possible candidate for lung cancer screening. For each of the following three statements related to the decision-making in this consultation, please select the response that best describes your actions with this patient.

	Strongly disagree	Somewhat disagree	Neither agree nor disagree	Somewhat agree	Strongly agree
a. I explained the advantages and disadvantages of screening to my patient.	○	○	○	○	○
b. I helped my patient understand all the risks and benefits and details about screening.	○	○	○	○	○
c. My patient and I reached an agreement on how to proceed.	○	○	○	○	○

22. When you talk to patients about lung cancer screening, how often do you encounter the following?

	Always	Usually	Sometimes	Never
a. Not having enough time to discuss screening with my patients	○	○	○	○
b. Patients who do not want to discuss lung cancer screening	○	○	○	○
c. Patients who have difficulty understanding the information I present about lung cancer screening	○	○	○	○
d. Patients who are unaware of lung cancer screening	○	○	○	○
e. Patients who do not perceive lung cancer as a serious health threat	○	○	○	○
f. Patients who cannot afford or lack adequate insurance coverage for lung cancer screening	○	○	○	○
g. Other	○	○	○	○

Please specify: (If endorsed "Always", "Usually", or "Sometimes" for g. Other above)

23. How comfortable are you advising patients about the benefits and risks of lung cancer screening?

◯ Very comfortable
◯ Somewhat comfortable
◯ Somewhat uncomfortable
◯ Very uncomfortable

Cervical Cancer Screening

24. When you talk to patients about cervical cancer screening, how often do you encounter the following?

	Always	Usually	Sometimes	Never
a. Not having enough time to discuss screening with my patients	◯	◯	◯	◯
b. Patients who do not want to discuss cervical cancer screening	◯	◯	◯	◯
c. Patients who have difficulty understanding the information I present about cervical cancer screening	◯	◯	◯	◯
d. Patients who are unaware of cervical cancer screening	◯	◯	◯	◯
e. Patients who do not perceive cervical cancer as a serious health threat	◯	◯	◯	◯
f. Patients who cannot afford or lack adequate insurance coverage for cervical cancer screening	◯	◯	◯	◯
g. Other	◯	◯	◯	◯

Please specify: (If endorsed "Always", "Usually", or "Sometimes" for g. Other above)

HPV Vaccination

25. When you talk to patients about HPV vaccination, how often do you encounter the following?

	Always	Usually	Sometimes	Never
a. Not having enough time to discuss HPV vaccination with my patients	◯	◯	◯	◯
b. Patients who do not want to discuss HPV vaccination	◯	◯	◯	◯
c. Patients who have difficulty understanding the information I present about HPV vaccination	◯	◯	◯	◯
d. Patients who are unaware of HPV vaccination	◯	◯	◯	◯
e. Patients who do not perceive HPV as a serious health threat	◯	◯	◯	◯
f. Patients who cannot afford or lack adequate insurance coverage for HPV vaccination	◯	◯	◯	◯
g. Other	◯	◯	◯	◯

Please specify: (If endorsed "Always", "Usually", or "Sometimes" for g. Other above)

26. How important do you feel it is to encourage HPV vaccination for females age 9-17?

◯ Very important
◯ Somewhat important
◯ Somewhat unimportant
◯ Very unimportant

27. How important do you feel it is to encourage HPV vaccination for females age 18-26?

◯ Very important
◯ Somewhat important
◯ Somewhat unimportant
◯ Very unimportant

28. How important do you feel it is to encourage HPV vaccination for males age 9-17?

◯ Very important
◯ Somewhat important
◯ Somewhat unimportant
◯ Very unimportant

29. How important do you feel it is to encourage HPV vaccination for males age 18-26?

◯ Very important
◯ Somewhat important
◯ Somewhat unimportant
◯ Very unimportant

30. Does your main clinic have a mechanism to remind you or other members of the care team that a patient is due for cancer screening? (CHECK ALL THAT APPLY).

☐ Yes, special notation or flag in patient's chart
☐ Yes, computer prompt or computer-generated flow sheet
☐ Yes, I routinely look it up in the medical record at the time of a visit
☐ Yes, other mechanism
☐ No, not that I have noticed
☐ No

31. Is there anything you think we should know that can help us better understand and/or improve what Essentia Health does to help you and your staff better manage your patients' cancer prevention needs?
